# Simultaneous tracking of psychophysical detection thresholds and evoked potentials to study nociceptive processing

**DOI:** 10.3758/s13428-019-01338-7

**Published:** 2020-01-21

**Authors:** Boudewijn van den Berg, Robert J. Doll, Alexander L.H. Mentink, Pieter S. Siebenga, Geert J. Groeneveld, Jan R. Buitenweg

**Affiliations:** 1 Biomedical Signals and Systems, Technical Medical Centre, University of Twente, PO Box 217, 7500 AE Enschede, The Netherlands; 2 Centre for Human Drug Research, Leiden, the Netherlands

**Keywords:** Evoked potential, Nociceptive processing, Linear mixed regression, Generalized linear mixed regression, Psychometric curve, Nociceptive detection threshold, Threshold tracking

## Abstract

Measuring altered nociceptive processing involved in chronic pain is difficult due to a lack of objective methods. Potential methods to characterize human nociceptive processing involve measuring neurophysiological activity and psychophysical responses to well-defined stimuli. To reliably measure neurophysiological activity in response to nociceptive stimulation using EEG, synchronized activation of nerve fibers and a large number of stimuli are required. On the other hand, to reliably measure psychophysical detection thresholds, selection of stimulus amplitudes around the detection threshold and many stimulus–response pairs are required. Combining the two techniques helps in quantifying the properties of nociceptive processing related to detected and non-detected stimuli around the detection threshold.

The two techniques were combined in an experiment including 20 healthy participants to study the effect of intra-epidermal electrical stimulus properties (i.e. amplitude, single- or double-pulse and trial number) on the detection thresholds and vertex potentials. Generalized mixed regression and linear mixed regression were used to quantify the psychophysical detection probability and neurophysiological EEG responses, respectively.

It was shown that the detection probability is significantly modulated by the stimulus amplitude, trial number, and the interaction between stimulus type and amplitude. Furthermore, EEG responses were significantly modulated by stimulus detection and trial number. Hence, we successfully demonstrated the possibility to simultaneously obtain information on psychophysical and neurophysiological properties of nociceptive processing. These results warrant further investigation of the potential of this method to observe altered nociceptive processing.

## Introduction

Identification of malfunctioning mechanisms in the nociceptive system of chronic pain patients is challenging, as it is difficult to quantify properties of nociceptive processing. Many investigators aim to observe properties of central nociceptive processing by measuring neurophysiological responses to cutaneous nociceptive stimuli. The processing of nociceptive stimuli can be divided into three steps: 1) activation of peripheral nociceptive nerve fibers by a stimulus, 2) processing of peripheral input into various central neural representations leading, to 3) conscious detection, evaluation, and subsequent actions (Dehaene & Naccache, 2001). Observing brain responses related to these steps in nociceptive processing could be useful to study the enhanced pain sensation in chronic pain patients, as plausible explanations include altered mechanisms of upstream nociceptive processing (Sandkühler, 2009) as well as altered brain mechanisms involving pain perception and regulation (Apkarian, Bushnell, Treede, & Zubieta, 2005). Therefore, to be able to study such changes in nociceptive processing using neurophysiological responses, a major step would be to establish methods to quantify stimulus–response relations.

If one wants to specifically study the nociceptive system, stimuli should activate nociceptive nerve fibers, i.e. Aδ and C fibers, selectively (K. Inui & Kakigi, 2011). One method which preferentially activates nociceptive nerve fibers is intra-epidermal electric stimulation (Koji Inui, Tran, Hoshiyama, & Kakigi, 2002; Otsuru et al., 2009; Otsuru et al., 2010). To preferentially activate nociceptive nerve fibers using intra-epidermal electrical stimulation, one has to apply stimulus intensities below twice the detection threshold (A. Mouraux, Iannetti, G. D., & Plaghki, L., 2010).

Changes in the detection threshold, for example drifting (Fründ, Haenel, & Wichmann, 2011), can be observed using adaptive psychophysical methods (Doll, Veltink, & Buitenweg, 2015). Recently, a method was developed for simultaneously tracking the nociceptive detection threshold (NDT) for multiple properties of intra-epidermal electric stimuli, such as the number of pulses and the inter-pulse interval. Simultaneously observing the NDTs for different sets of stimulus properties can be used to characterize various aspects of nociceptive processing in terms of psychophysical (detection) thresholds (i.e. NDT) and slopes (i.e. the gradient of detection probability at the detection threshold). Using this method, the drift over time of the NDT and the slope and variation of the detection probability with respect to the number of pulses, inter-pulse interval, and pulse width was quantified (Doll, Maten, Spaan, Veltink, & Buitenweg, 2016). By tracking the NDT, it is possible to observe modulation of nociceptive processing, e.g. the effect of diffuse noxious inhibitory control by tracking NDTs during a cold pressor test (Doll, Buitenweg, Meijer, & Veltink, 2014) and an altered NDT up to several weeks after the application of capsaicin (Doll, van Amerongen, et al., 2016). The slope provides additional information about the reliability of stimulus detection by subjects (Gold & Ding, 2013). However, a major limitation of this tracking technique is that it is unknown whether characteristics of the detection probability are related to psychological or physiological factors. For example, a changing NDT could be interpreted as either a changing subjective detection criterion, or as neuroplasticity of nociceptive processing.

More specific insights into nociceptive processing might be obtained by measuring neurophysiological activity in response to a stimulus using electroencephalography (EEG), as this activity is thought to be related to the various central neural representations of the stimulus that contribute to stimulus perception (A. Mouraux, Iannetti, Baumgärtner, & Treede, 2015). It was recently shown that the amplitude of temporal components of the evoked potentials in response to suprathreshold intra-epidermal electrical stimulation can be used to observe altered central processing, such as central sensitization induced by capsaicin (Liang, Lee, O’Neill, Dickenson, & Iannetti, 2016) and by high-frequency stimulation of the skin (Manresa, Andersen, Mouraux, & van den Broeke, 2018). Recent studies demonstrate that the effect of stimulus properties on the evoked potential can be successfully quantified using linear mixed regression (LMR) (Van den Berg & Buitenweg, 2018; C. J. Vossen, Vossen, Marcus, Van Os, & Lousberg, 2013; H. G. Vossen, Van Breukelen, Hermens, Van Os, & Lousberg, 2011). Vossen et al. showed the potential of this technique to identify altered nociceptive processing by demonstrating altered habituation of the evoked potential to nociceptive stimuli in patients with chronic low-back pain (C. J. Vossen, Vossen, Joosten, Van Os, & Lousberg, 2015).

Acquiring EEG during multiple threshold tracking allows for simultaneous observation of detection thresholds and corresponding evoked potentials in response to multiple types of stimuli while targeting nociceptive pathways by stimulating around the NDT. As such, combining the two methods in a single experiment might create an improved method for observing (altered) nociceptive processing. Therefore, the aim of this work is to study the potential of this combination of techniques to explore how stimulus properties are reflected in NDTs and vertex potentials in healthy subjects.

Similar to earlier studies, detection probability and the corresponding NDT are tracked in response to intra-epidermal stimuli. Responses to intra-epidermal stimuli with properties similar to those studies (Doll, Maten, et al., 2016; Doll, van Amerongen, et al., 2016) are analyzed using generalized mixed regression to observe whether the effects of stimulus properties on the detection probability can be replicated. In addition, this study combines EEG responses with the existing method. A method for integrated analysis of those responses and the detection probability is outlined, in which LMR is used to explore whether the evoked potentials observed during this experiment correlate to stimulus properties.

## Methods

The data presented in this paper are acquired from a larger set involving various psychophysical and neurophysiological recordings with stimulation at multiple skin locations, performed on two occasions. For the purpose of this paper, the current data set involves recordings obtained during the first occasion. It contains EEG and stimulus–response pair data collected at three different skin locations: two adjacent locations on the participant’s right forearm and one location on the left forearm.

### Participants

After approval by the Medical Review and Ethics Committee (Foundation BEBO, Assen, the Netherlands) and in accordance with the Declaration of Helsinki, 20 healthy male participants were enrolled after providing written informed consent. Inclusion criteria were as follows: age 18–65 years, body mass index between 19 and 30 kg m^−2^, and good medical condition defined as absence of clinically significant findings in their medical history, physical examination, and vital signs. Exclusion criteria were illicit drug use, frequent caffeine use (> 8 units per day), smoking (> 10 cigarettes per day), extreme response to capsaicin 1% topical cream (Numeric Rating Scale > 8 out of 10), skin abnormalities, and abnormal blood pressure. In a separate part of the study (not reported here), erythema or reddening of the skin was measured. As this cannot be measured in dark-toned skin, participants with dark-toned skin (Fitzpatrick scale types V and VI) were excluded from the study. The use of over-the-counter medication within 3 days of measurements was not allowed. During the study, participants were to refrain from strenuous physical exercise and the use of all (methyl)xanthenes and alcohol. Participants unable to tolerate the assessments at screening were excluded. Participants received remuneration for participation and could withdraw at any time without jeopardizing the remuneration.

### Stimuli

**Fig. 1:**
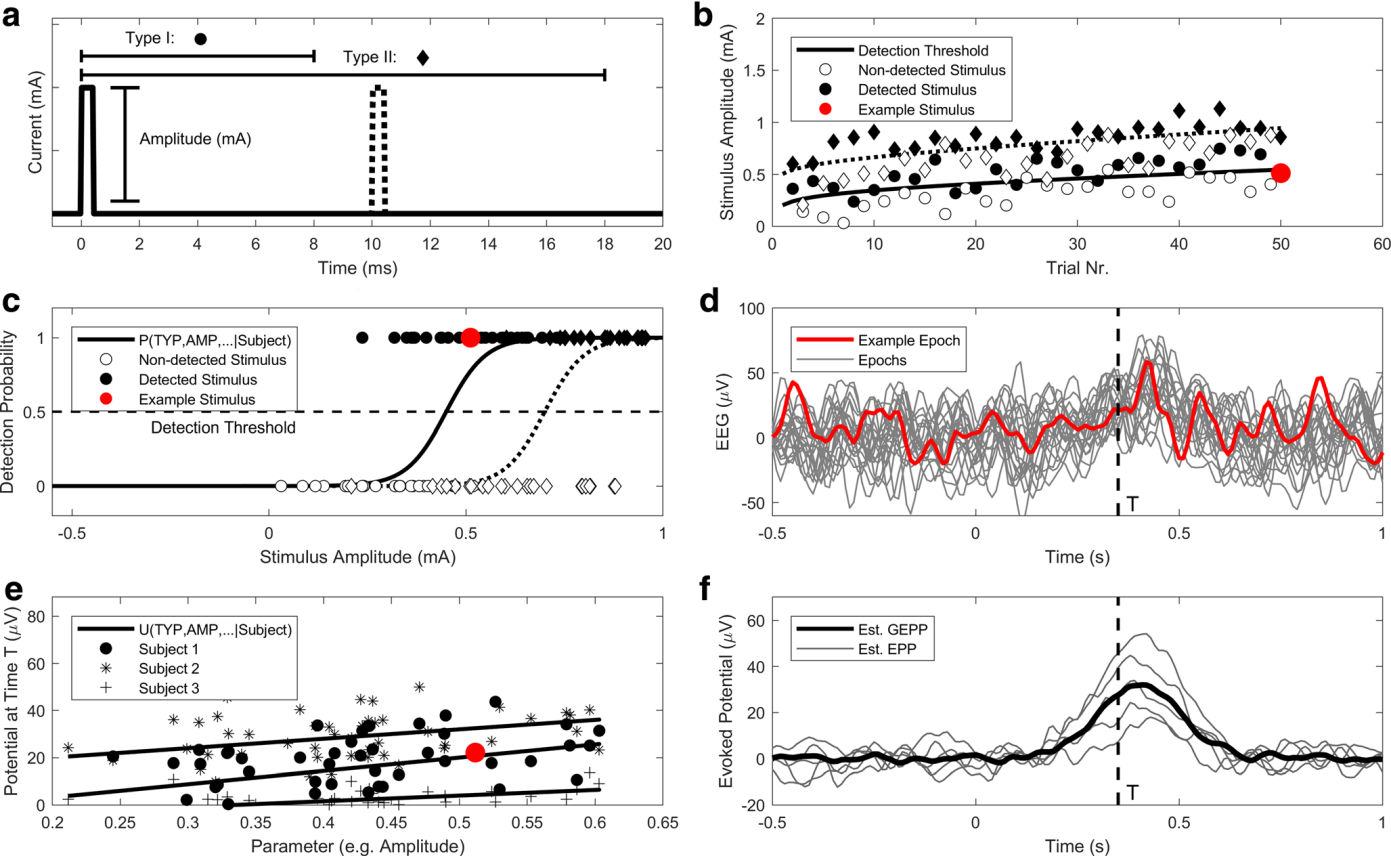
Workflow for simultaneous recording and analysis of NDTs and EEG. **a** Stimulate using one or more types of phasic stimuli. **b** Use an adaptive paradigm to stimulate close to the detection threshold and record whether a stimulus is detected. **c** Use GLMR to compute the detection probability (P) as a function of stimulus properties, e.g. the stimulus type (TYP) and amplitude (AMP), and determine the detection threshold with respect to every stimulus. **d** Record an EEG epoch for every stimulus. **e** For every time point T in the set of EEG epochs, use LMR to compute the potential (U) as a function of stimulus properties, e.g. the stimulus type (TYP) and amplitude (AMP). **f** Use the obtained LMR for every point in time to predict the potential for a specific set of parameters over all subjects (grand evoked potential prediction, GEPP) or for specific subjects (evoked potential prediction, EPP). As an example, one stimulus and the corresponding results are shown in red

Participants were presented with intra-epidermal electrical stimulation on the anterior part of the left and right volar forearm, to generate a pinprick-like sensation. The electrode for stimulation consisted of an array of five interconnected microneedles serving as a compound cathode. Needles protruded 0.2 mm into the skin, allowing for preferential nociceptive stimulation. A 50 × 90 mm transcutaneous electrical nerve stimulation (TENS) electrode served as the anode and was placed distally from the intra-epidermal electrode. Electric stimuli were administered using a custom-built constant current stimulator (NociTRACK AmbuStim, University of Twente, Enschede, the Netherlands). The electric stimuli were cathodic rectangular pulses with two different settings (Fig. 1a):

- A single 210 μs pulse
- A double 210 μs pulse with an inter-pulse interval of 10 ms

### Procedure

Participants were seated in a comfortable chair directed towards a wall and were asked to focus on one point on the wall. The procedure started by probing the initial detection threshold with a normal staircase procedure and a step size of 0.05 mA. Subsequently, stimulus amplitudes were chosen according to an adaptive staircase procedure enabling stimulation to be performed near the detection threshold (Doll et al., 2015), as is illustrated in Fig. 1b. A set of seven equidistant stimulus amplitudes centered around the detection threshold was defined, from which the upcoming stimulus was randomly selected. All amplitudes in the set were increased and decreased with a fixed step size of 0.05 mA after a non-detected stimulus and detected stimulus, respectively. Single-pulse and double-pulse stimuli were presented in random order.

During the application of those stimuli, participants were instructed to hold a response button until stimulus detection. While non-detected, the stimulator continued to apply stimuli with a randomized inter-stimulus interval ranging from 3 to 10 s. After a stimulus was detected, participants were to release the button and then press the button again after about 1 s. This procedure was repeated until 100 stimulus-response pairs (50 per stimulus type) were collected per skin location (approximately 10 min. per location). Participants had a small break when the electrode was moved to a different location. A custom computer program (written in LabVIEW 2011, SP1) controlled all stimulation procedures, as well as the registration of stimulus amplitudes in mA, stimulation times in milliseconds, and responses to stimuli (i.e. detected or non-detected).

### Electroencephalography

EEG was recorded at a sample frequency of 1024 Hz with a Refa amplifier (TMSi B.V., Oldenzaal, the Netherlands) using a 24-channel electrode cap (10/20 layout and mastoids) and additional leads on the earlobes with the common average as reference. Eye movements were recorded using bipolar electrodes placed at about 2 cm superior to the right eye outer canthus and 2 cm inferior to the left eye outer canthus. Electrode impedances were kept below 5 kΩ.

EEG data were preprocessed using FieldTrip (Oostenveld, Fries, Maris, & Schoffelen, 2011). For each trial of the experiment, which is defined as the application of one stimulus, EEG epochs were extracted from 0.5 s pre- to 1 s post-stimulus (Fig. 1d). Epochs were bandpass-filtered between 0.1 and 30 Hz using a second-order Butterworth high-pass filter and a sixth-order Butterworth low-pass filter, and baseline-corrected using the 500–0 ms pre-stimulus interval. Epochs containing eye blink artefacts were removed using automatic rejection of epochs containing potentials larger than three times the standard deviation based on either the electrooculography (EOG) or FPz channel. Stimulus–response pairs corresponding to those epochs were also removed from the data set prior to statistical analysis.

### Statistical analysis

Data preparation and statistical analysis of the effects of stimulus properties on EPs was performed in MATLAB 2017b (MathWorks, Inc.). Statistical analysis of the effect of stimulus properties on the detection probability was performed in R using the lme4 toolbox (Bates, Mächler, Bolker, & Walker, 2015).

#### Effect of stimulus properties on detection probability

The effect of stimulus properties on the detection probability was evaluated using generalized linear mixed regression (GLMR) using a logit link function. The intercept, stimulus amplitude (*AMP*, in mA), type (*TYP*), trial number (*TRL*), stimulation location (*LOC*), and the interaction between the stimulus amplitude and stimulus type were included as fixed effects. First, all fixed effects were included as random effects, grouped by subject. Subsequently, random effects were excluded if this led to a lower model Akaike information criterion (AIC). As a result, between-subjects random effects were included for the intercept, the stimulus amplitude, stimulus type, trial number, and location. The trial number variable was centered and scaled prior to analysis to speed up the estimation process. An unstructured covariance matrix was used to model the random effects. The equation of the GLMR model used, in Wilkinson notation, is shown in (1).

1 $$ \mathit{\ln}\left(\frac{P_d}{1-{P}_d}\right)\sim 1+ AMP\ast TYP+ TRL+ LOC+\left(1+ AMP\ast TYP+ TRL+ LOC|S\right) $$

Type III Wald Chi-square statistics were used to test the main and interaction effects of the fixed effects. Confidence intervals of the regression parameters were based on the Wald *z* statistics. Threshold and slope estimates were obtained from the regression parameters. The logistic psychophysical curves representing the detection probability were computed by inverse-logit transformation of the regression parameters.

#### Effect of stimulus properties on EEG

EEG data at the Cz-A1A2 derivation were used for modeling and analysis. The effect of stimulus properties on the EEG potential (*U*_*EEG*_) was computed for every point in time using LMR (Fig. 1e). The equation of the LMR model used, in Wilkinson notation, is shown in (2).

2 $$ {U}_{EEG}\sim 1+ AMP\ast TYP\ast RES+ TRL\ast RES+ LOC\ast RES+\left(1+ AMP+ RES+ TRL|S\right) $$

The stimulus amplitude (*AMP*, in mA), stimulus type (*TYP*), trial number (*TRL*), and the interaction between stimulus amplitude and stimulus type were included as fixed effects in the LMR model to study the effect of these parameters. Stimulation location (*LOC*) was also included to prevent potential confounding by the location or the order in which the locations were measured. An interaction of all those fixed effects with response (*RES*) as well as the main effect of response were included to account for differences in processing between detected and non-detected stimuli. Random effects grouped by subject (*S*) were chosen by first including all fixed effects and interactions, and subsequently excluding random effects if this led to a lower model AIC. An unstructured covariance matrix was used to model the random effects.

For every point in time, regression parameters were estimated by optimization of the restricted maximum likelihood. Normality of the model residuals was assessed by computing residual skewness and kurtosis along the entire epoch. The significance of the fixed effects in the LMR model was tested using the *t* statistic with Satterthwaite’s method for estimating the degrees of freedom. Significance values were then corrected for positive dependence in time using the Benjamini-Hochberg correction (Groppe, Urbach, & Kutas, 2011; Hochberg & Benjamini, 1995). LMR parameters were used to model the effect of trial parameters on the time-locked EEG, resulting in an evoked potential prediction (EPP) on the subject level and a grand evoked potential prediction (GEPP) on the group level (Fig. 1f). This was done by filling out the LMR equation for each point in time using the corresponding trial parameters.

## Results

A total of 20 participants participated in the experiment. One participant withdrew from the experiment and was replaced. Of all the measurements, seven were excluded due to technical issues, leaving 53 measurements available for analysis. After rejecting epochs contaminated with ocular activity, 216 ± 52 stimulus response pairs and corresponding EEG epochs were available per participant, in which 90.5% of the stimuli were estimated to be below two times the NDT.

### Effect of stimulus properties on detection probability

**Fig. 2:**
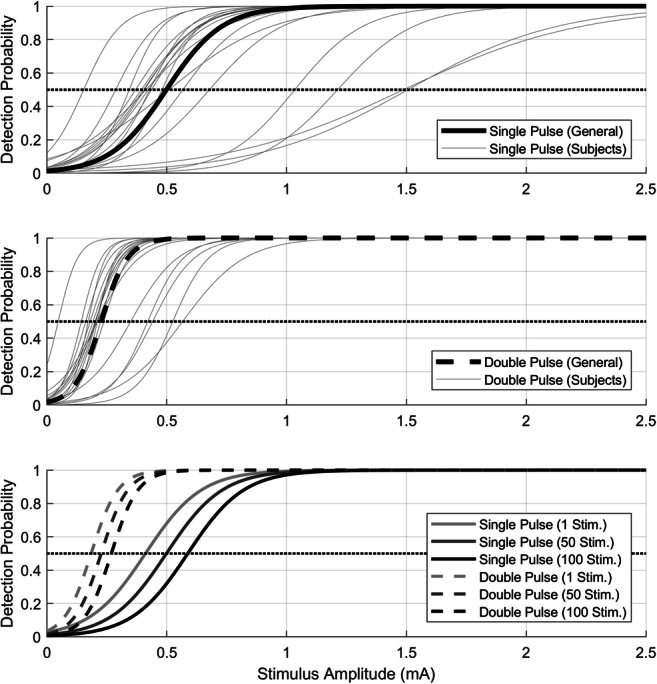
Logistic psychophysical curves modeling the detection probability were obtained by inverse-logit transformation of the regression parameters. The resulting general and subject-level detection probabilities for single- and double-pulse stimuli are shown in the first two figures, and their variation with respect to the trial number is shown in the bottom figure. The first two figures show that the psychophysical curve of double-pulse stimuli has a steeper slope and a lower NDT than the curve for single-pulse stimuli. The bottom figure shows that the NDT increases with respect to the number of trials, where ‘1 Stim.’ corresponds to the start, ‘50 Stim.’ to the middle, and ‘100 Stim.’ to the end of the experiment

Table 1 presents the estimated log-odds for the parameters of the GLMR model with corresponding 95% confidence intervals and significance tests for the main effects. Stimulus amplitude, trial number, and the interaction between stimulus type and amplitude had a significant effect on the detection probability. However, there was no indication of the stimulation location and stimulus type having a significant effect on the intercept of the detection probability. The regression parameters were inverse-logit-transformed to obtain the logistic psychophysical curves for both settings at the first stimulation area (Fig. 2). The average NDTs for a single- and double-pulse stimulus were 0.50 and 0.22 mA, respectively. The slopes for single- and double-pulse stimuli were found to be 8.55 and 17.93 mA^−1^, respectively. Adding a second pulse to the stimulus significantly decreased the NDT and significantly increased the detection slope (*p* < 0.001). The NDT increased with respect to the trial number. Effectively, the NDT for single-pulse stimuli increased from 0.41 mA at the start of the experiment to 0.59 mA at the end of the experiment, while the NDT for double-pulse stimuli increased from to 0.18 mA to 0.27 mA.

**Table 1:** Regression parameter estimates of the fixed effects of the GLMR, corresponding confidence intervals, and type III Wald statistics of the main effects. There is a significant increase in detection probability with respect to amplitude. There is a significant decrease in the detection probability with respect to the trial number, which results in an increase in the NDT. Furthermore, there is a significant positive interaction between stimulus type and amplitude, effectively resulting in a higher detection probability and lower NDT for double-pulse stimuli

Parameter	Parameter estimate	95 % Confidence interval	Effect χ^2^(df)	Effect *p*
(Intercept)	−3.52	[−4.36 −2.68]	67.66 (1)	< .001
Stimulus amplitude	8.55	[ 6.78 10.32]	89.67 (1)	< .001
Stimulus type			0.39 (1)	.53
Double pulse	0.21	[−0.45 0.88]		
Location			3.18 (2)	.20
Second	−0.72	[−1.70 0.26]		
Third	0.12	[−1.04 1.28]		
Trial number	−0.42	[−0.58 −0.27]	27.58 (1)	< .001
Type × Amplitude			57.94 (1)	< .001
Double pulse	9.38	[ 6.96 11.79]

### Effect of stimulus properties on evoked potentials

**Fig. 3:**
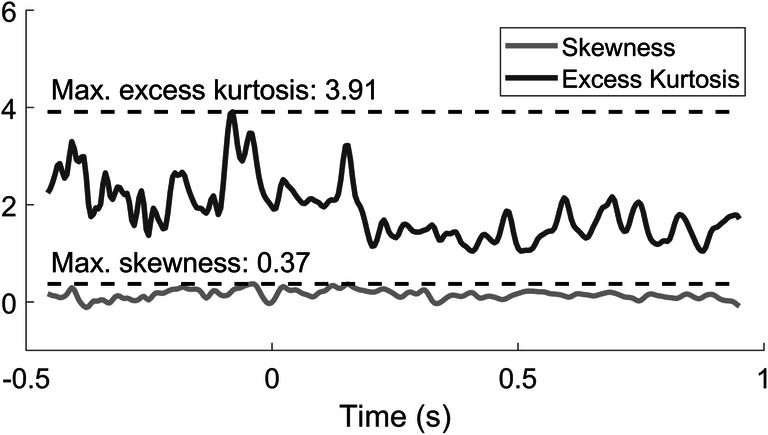
Skewness and excess kurtosis of LMR residuals over time

To determine the effect of stimulus parameters on the evoked potential, LMR parameters were computed for every point in time at Cz-A1A2 based on the entire set of EEG epochs. The skewness and excess kurtosis of the model residuals along the entire epoch are shown in Fig. 3. The excess kurtosis has a maximum value over the entire epoch of 3.91, while the skewness has a maximum value over the entire epoch of 0.37.

**Fig. 4:**
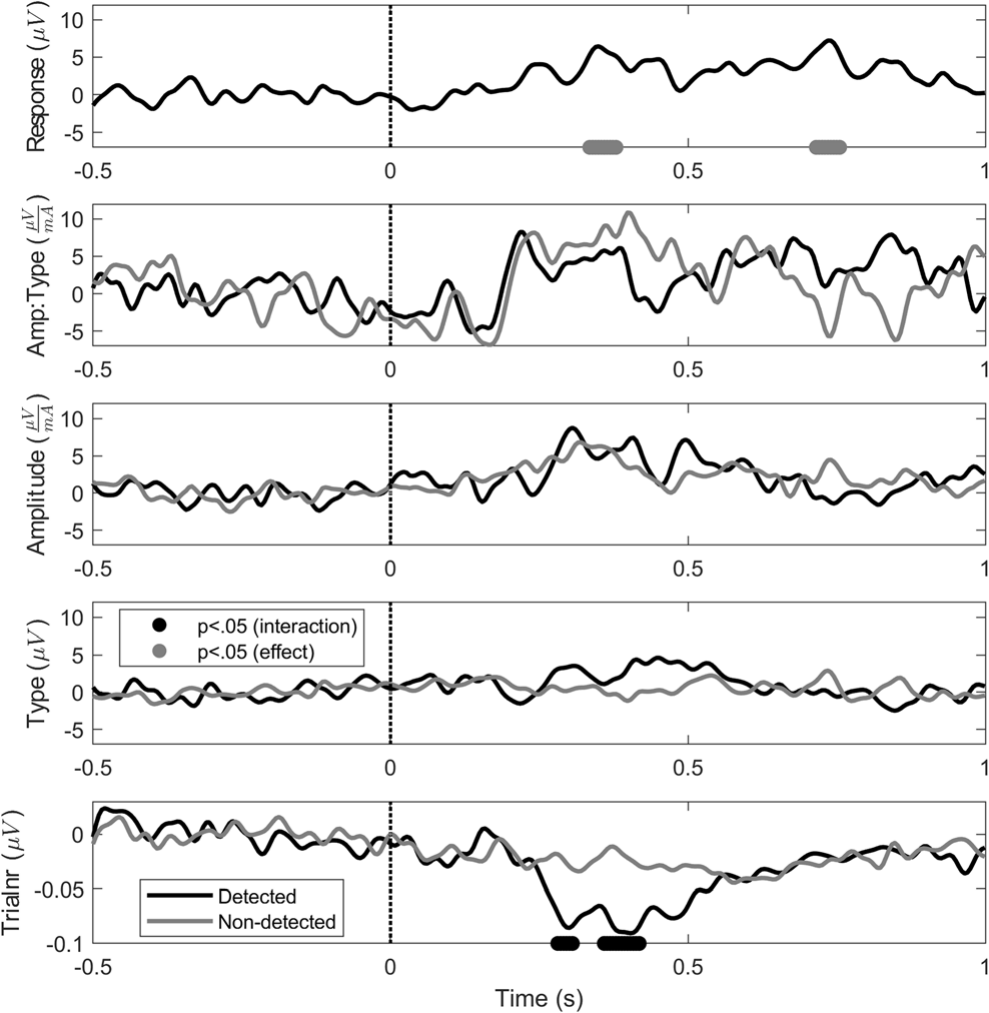
LMR parameters over time, which quantify the effect of each stimulus property on the evoked potential for detected (black) and non-detected (gray) stimuli. Gray and black dots below the curves respectively indicate an effect and an interaction with response that is significantly different from zero (*p* < 0.05)

Parameters and their significances are shown in Fig. 4. Parameters of detected and non-detected stimuli appear to show a positive effect of amplitude, type, and the interaction between amplitude and type on the evoked potential. However, none of these effects or their interactions with response is significant. A negative effect of trial number on the evoked potential was observed. This effect significantly interacts with response between 250 and 500 ms, as the effect of trial number is much larger for detected stimuli at these latencies.

**Fig. 5:**
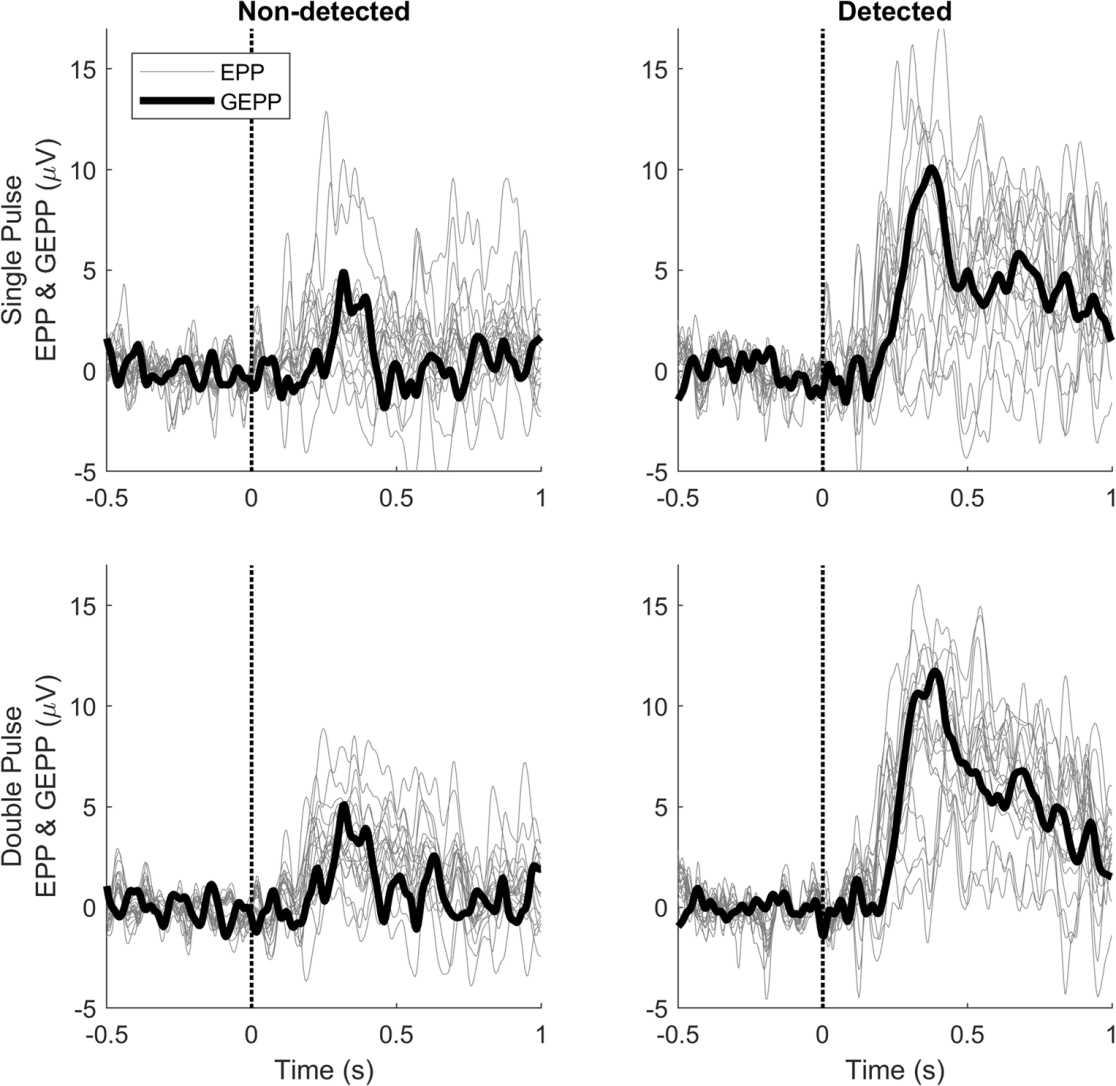
GEPPs and EPPs at Cz-A1A2 in response to stimuli with a stimulus amplitude equal to the NDT, computed using LMR. EPPs (gray) show the spread of subject responses, which is at its largest around 340 ms. GEPPs show the responses on group level, with a peak around 340 ms and a late positive component between 500 and 1000 ms

Figure 5 shows the GEPP and EPPs of single-pulse and double-pulse detected and non-detected stimuli. For detected and non-detected stimuli, there was a positive peak in the GEPP around 340 ms, and a late positive component between 500 and 1000 ms. The peak at 340 ms had a larger amplitude for detected stimuli (single pulse: 10.10 μV, double pulse: 11.75 μV) than for non-detected stimuli (single pulse: 4.89 μV, double pulse: 5.10 μV). Furthermore, there was a larger P340 amplitude for double-pulse stimuli than for single-pulse stimuli.

**Fig. 6:**
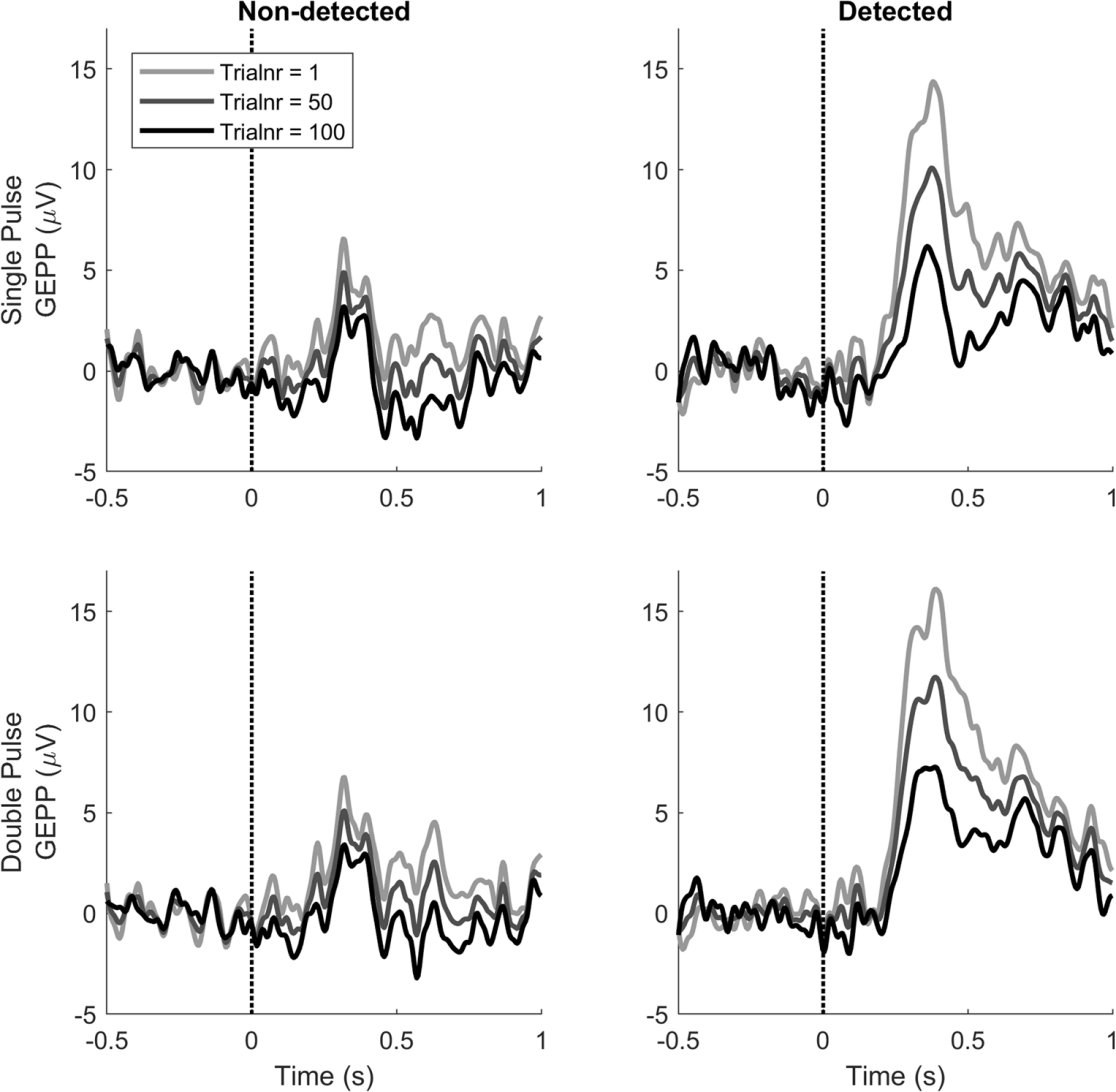
GEPPs in response to stimuli with a stimulus amplitude equal to the NDT after 1, 50, and 100 trials, computed using LMR. The GEPP varies the most around 340 ms with respect to detected stimuli

The effect of trial number on the GEPP is displayed in Fig. 6. GEPPs corresponding to both detected and non-detected stimuli are decreasing with an increasing number of trials, with a major decrease between 200 ms and 900 ms. For detected stimuli, the P340 amplitude decreases from 14.37 μV at the first trial to 6.18 μV at the last trial (single-pulse) and from 16.12 μV at the first trial to 7.27 μV at the last trial (double-pulse). For non-detected stimuli, the P340 amplitude decreases from 6.56 μV at the first trial to 3.18 μV at the last trial (single-pulse) and from 6.77 μV at the first trial to 3.41 μV at the last trial (double-pulse).

**Fig. 7:**
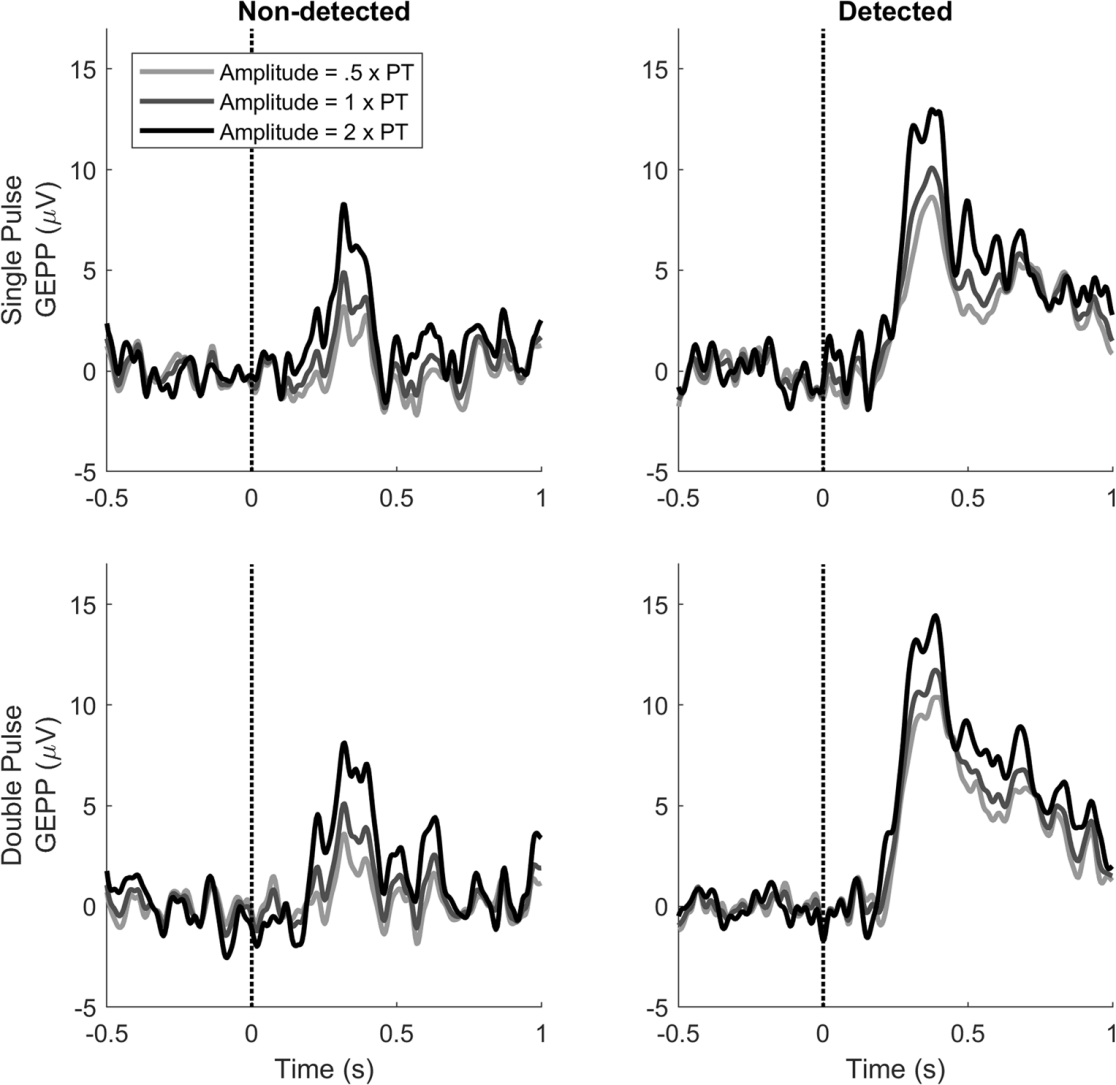
GEPPs in response to stimuli with a stimulus amplitude of 0.5, 1.0, and 2 times the NDT. The GEPP varies the most around 340 ms for both detected and non-detected stimuli

The effect of stimulus amplitude on the GEPP is shown in Fig. 7. Both non-detected and detected stimuli show some variation with respect to the stimulus amplitude. However, the GEPP in response to detected stimuli is larger than the GEPP in response to non-detected stimuli, regardless of using the same stimulus amplitude. For example, the amplitude of the GEPP for detected stimuli at 2 times the NDT is 12.98 μV (single-pulse) and 14.45 μV (double-pulse), while for non-detected stimuli at 2 times the NDT, this is 8.28 μV (single-pulse) and 8.12 μV (double-pulse).

## Discussion

In this experiment, evoked potentials and NDTs with respect to nociceptive specific stimuli were measured simultaneously. The detection probability was tracked using an adaptive stimulus selection procedure aimed at stimulating close to the NDT, while EEG was recorded in response to every stimulus. Here, the same electrode was used, and the effect of temporal stimulus properties on the NDT was quantified using the same method to track the detection threshold (Doll, Maten, et al., 2016). The computed NDTs and slopes in this experiment are in a similar range to those reported earlier by Doll et al.

The evoked potentials were acquired and analyzed in combination with stimulus–response pairs. LMR was used to compute the effects of stimulus properties on the evoked potential. Evoked potentials in response to detected and non-detected stimuli were observed in the GEPPs and EPPs, as shown in Figs. 5, 6, and 7. The evoked potential in response to detected stimuli shows a positive component with the peak amplitude near 340 ms (i.e. P340), with a late positive component between 500 and 1000 ms.

The waveform and latency of the observed P340 appears to correspond to the P2 reported in literature (Legrain, Bruyer, Guérit, & Plaghki, 2003; Legrain, Guérit, Bruyer, & Plaghki, 2002). The P340 will be referred to as the P2 in the next sections. While others report a positive correlation between the stimulus amplitude and the P2 amplitude (Ohara, Crone, Weiss, Treede, & Lenz, 2004), this correlation was not significant here. As the detection threshold increased with the number of given stimuli, the stimulation amplitudes also increased (see Fig. 2). As a result, the given stimulus amplitudes were only varied within a relatively small range around the detection threshold, limiting the observability of this correlation. Regardless, a trend suggesting an increased P2 amplitude with increased stimulus amplitudes can be visually observed (see Fig. 7). Potential correlation could be more accurately assessed in follow-up studies by increasing the range of stimulus amplitudes or by increasing the number of trials (see also Section 4.2).

The observed late positive component between 500 and 1000 ms (see Fig. 7) was significantly different between detected and non-detected stimuli. The behavior and the latency of this component are similar to earlier reports of the P3b component (Legrain et al., 2003). Although this component is normally evoked by rare stimuli in an oddball paradigm, this component has also been obtained in one-stimulus paradigms where subjects have to detect all stimuli (Polich, Eischen, & Collins, 1994). Based on these similarities with the literature, the late positive component between 500 and 1000 ms will be referred to as the P3b in the next sections.

### Effect of stimulus properties on detection probability and evoked potentials

The effect of stimulus properties on the evoked potentials was studied by computing the GLMR and LMR parameters in Table 1 and Fig. 4, and predicting the GEPPs and EPPs in Figs. 5, 6, and 7. In general, it can be observed that all GEPPs increase in amplitude when a stimulus is detected. The LMR parameter for response in Fig. 4 shows that parts of both the P2 and P3b are significantly modulated by stimulus detection. While the P3b appears to be exclusively present when a stimulus was detected, the P2 can be identified in responses to both detected and non-detected stimuli. This observation could mean that the P2 component contains information about an internal stimulus representation prior to the conscious detection of stimuli. However, it is important to note that this component could also be present due to false-negative responses of the subjects, in which subjects did feel the stimulus but did not release the response button.

When comparing the NDTs and slopes corresponding to the single- and double-pulse stimuli, it was observed that the NDT was lower and the slope was steeper for a double-pulse stimulus than for a single-pulse stimulus (see Fig. 2). This is similar to earlier findings (Doll, Maten, et al., 2016). There, it was hypothesized that a facilitating nociceptive mechanism is also involved in the processing of double pulses, resulting in an even lower NDT for double-pulse stimuli than would be expected based on the principle of probability summation. The underlying physiology of this effect remains unknown and could include central (e.g. temporal summation, short-term synaptic plasticity [Zucker & Regehr, 2002]), or peripheral (e.g. subthreshold or suprathreshold super-excitability [Bostock et al., 2005]) mechanisms. Even though such a difference was observed in NDT and slopes, no significant difference in P2 amplitudes for single- and double-pulse stimuli was observed here (see Fig. 4). Possible explanations for this are discussed in Section 4.2. Regardless, the differences in nociceptive processing between single-pulse and double-pulse stimuli might be further studied in future work combining this technique with experimental pain models to study the individual contributions of specific inhibitory and facilitating mechanisms to this effect.

In Fig. 6, it is shown that the GEPP around the P2 decreases with respect to the trial number. This effect can be observed most clearly on the GEPP in response to detected stimuli. Figure 4 shows that there is indeed a negative effect of trial number on the evoked potential, with a significant interaction with response. A similar effect is observed for the detection probability in Fig. 2 and a significant negative parameter for the effect of trial number on detection probability in Table 1. Such a negative effect of the number of received stimuli was shown in earlier studies on the evoked potential by Vossen et al. (C. J. Vossen et al., 2013; H. G. Vossen et al., 2011) and on the NDT and detection probability by Doll et al. (Doll, Maten, et al., 2016). In both studies, this effect was referred to as habituation. This effect might be attributed to decreasing attention to the stimuli or to a changing criterion for stimulus detection. Although the exact mechanism is unknown, estimating the effect of trial number on the evoked potential and the NDT is a relevant subject for further studies, as it could be a potential biomarker for several types of diseases, such as chronic low back pain (C. J. Vossen et al., 2015), fibromyalgia (Smith et al., 2008), and migraine (Valeriani et al., 2003).

In Fig. 7, an increase in the GEPP with respect to stimulus amplitude is shown, corresponding to a positive LMR parameter for stimulus amplitude and the interaction between stimulus amplitude and stimulus type in Fig. 4. As both parameters were non-significant, their true effect size for both detected and non-detected stimuli is to be evaluated in future studies. Recommendations for future studies for more accurate measurements of these effects are discussed in the next paragraph.

### Recommendations for simultaneous tracking of psychophysical detection thresholds and evoked potentials

This study was a first-time demonstration of the concept of simultaneous tracking of psychophysical detection thresholds and evoked potential. As no previous EEG data were available from such a study design, power considerations were not based on EEG data. Furthermore, no assumptions could be made regarding the evoked potential component latencies, and therefore no tests could be defined a priori. Instead, EEG data were tested in an exploratory fashion at every latency, requiring temporal correction for retesting using the Benjamini-Hochberg correction. Although this method guarantees that our false discovery rate will remain below the set critical value (5%), this could reduce the power of potentially significant effects. As the range of amplitudes was relatively limited, potential stimulus amplitude effects on the EP could not be found. If an effect exists, it might be observed by increasing the range of stimulus amplitudes. Furthermore, future studies might also improve the accuracy of estimated effect sizes by increasing the number of trials and subjects.

In addition, Fig. 3 shows a relatively high excess kurtosis (max. 3.91) of the LMR residuals. A high kurtosis indicates that the residual distribution has fat tails associated with outlying values of EEG potential, which results in a loss of power in the statistical tests. Such outliers are likely caused by artefacts in EEG data (Delorme, Sejnowski, & Makeig, 2007). As in this study a high kurtosis remains despite rejection of EOG artefacts, potential improvement could be made by either 1) improved training of participants to prevent EEG artifacts or 2) enhanced detection of artefacts in EEG data. Furthermore, excessive EEG noise might obscure any small variations in the evoked potential with respect to stimulus parameters. As the set of stimuli comprised a relatively narrow range of stimulus amplitudes around the detection threshold, EEG noise might have influenced the significance of this parameter.

## Conclusion

This study demonstrated a method for recording NDTs and evoked potentials in response to stimuli around those thresholds, and quantifying the effect of stimulus properties on those measures. Threshold tracking made it possible to observe the time-course of the NDT and to center stimulation around the detection threshold to preferentially stimulate nociceptive nerve fibers. As evoked potentials are related to the central neural representations underlying stimulus perception, they could help to more objectively assess properties of nociceptive processing in a clinical context. This study quantified the effect of stimulus properties on healthy subjects as a proof of principle. However, the most interesting application of this method would be to study how nociceptive processing is modulated by experimental pain models, therapeutic interventions, and most importantly, chronic pain. In the current study, the exact mechanisms of the observed effects of stimulus properties on evoked potentials remain unknown, and some of these effects were insignificant. Therefore, subsequent studies should focus on accurately documenting the effects of stimulus properties on NDTs and evoked potentials in healthy subjects, and comparing those to the effects under abnormal conditions. Besides shedding more light on nociceptive system behavior, this could be used to determine whether combined NDT and evoked potential measurement provides a valid metric for alterations of the nociceptive system associated with chronic pain.
